# Genome sequence analysis of nsp15 from SARS-CoV-2

**DOI:** 10.6026/97320630018432

**Published:** 2022-04-30

**Authors:** Niti Yashvardhini, Deepak Kumar Jha, Amit Kumar, Manjush Gaurav, Kumar Sayrav

**Affiliations:** 1 Department of Microbiology, Patna Women’s College, Patna, 800 001, Bihar, India; 2 Department of Zoology, S.M.P. Girls Degree College, Ballia, 277401, Uttar Pradesh, India; 3 Department of Botany, Patna University, Patna-800 005, Bihar, India; 4 Department of Chemistry, V.K.S. University, Ara-802301, Bihar India

**Keywords:** SARS-CoV-2, Pandemic, COVID-19, Nsp15, Mutation

## Abstract

SARS-CoV-2 (Severe Acute Respiratory Syndrome), a causative agent of COVID-19 disease created a pandemic situation worldwide. Nsp15 is a uridine specific endoribonuclease encoded by the genome of SARS-CoV-2. It plays important role in processing viral RNA and, thus evades the host immune system. Therefore, it is of interest to identify mutants of nsp15 amongst Asian SARS-CoV-2 isolates, where a total of 1795 mutations, from 7793 sequences of Asia submitted till 31st January 2022, amongst which A231V, H234Y, K109N, K259R and S261A mutations were found frequent. Hence, we report data on the predicted secondary structure of wild type form followed by hydropathy plot, physiochemical properties, Ramachandran plot, B-cell epitopes prediction and protein modeling of wild type and mutant of nsp15 protein. Data shows that nsp15 of SARS-CoV-2 is a pontential candidate for the development of vaccine to control the infections of SARS-CoV-2.

## Background:

SARS-CoV-2, a novel betacoronavirus, belonging to the family Coronaviridae and order nidovirales is an enveloped, positive-sense, largest known RNA virus (genome size∼30kb) and the causative agent of coronavirus disease 2019 (COVID-19) [1,2]. Coronavirus outbreak initiated from a small sea food market in Wuhan, China [3]. Although, several vaccines candidates are approved and are used on a mass scale, however the contagious nature of this virus are still a great threat to human beings whose ripple effect poses a huge human health crisis worldwide. As of 31st January 2022, confirmed cases of COVID-19 have been reported to be 450, 229 and 635, including 6,019,085 casualties, globally by WHO. As a consequence of multiple mutations this virus showed high rate of variability which enable them to evade host immunity [4]. Due to its ability to show high rate of mutations and poor fidelity of its RNA polymerase, these viruses shows greater antigenic variability [5, 6] and these high rates are greatly linked to virulence and evolution of this virus, traits prerequisite for viral adaptation [7].

The genome of SARS-CoV-2 comprises of 14 open reading frame (ORF) sequences which encode 29 proteins including 4 types of structural proteins; S (spike), E (envelope), M (membrane), and N (nucleocapsid) that are essential for the assembly of a complete virion particle. Among 16 nonstructural proteins, nsp15 contains uridylate specific endoribonuclease (EndoU) in the catalytic C-terminal domain [8-10]. as it has only been detected in Nidovirales, the virus EndoU was given the name nidovirus EndoU (NendoU) [11-13]. The high-resolution crystal structure of Nsp15 from SARS-CoV-2 was determined. The N-terminal oligomerization domain, the middle domain, and the C-terminal catalytic domain constitute Nsp15. In addition, crystal structural analyses revealed a hexameric configuration made up of two trimers with a peculiar spatial configuration [8,14-17]. NendoU hydrolyzes the phosphordiester bonds at uridine sites of single- and double-stranded RNA, with 2', 3'-cyclic phosphordiester and 5'-hydroxyl termini produced [18, 19]. The pattern recognition receptor MDA5 is used by the host innate immune system to recognize the polyU sequence in negative-sense viral RNA, which is copied from the polyA sequence in viral RNA [20-21]. To limit the build up of the polyU-containing sequence in cells and so dodge the innate immune response, nsp15 cleaves its own negative-sense RNA [20,22-24]. nsp15, for example, decreases viral RNA in host cells and hinders RNA-activated antiviral responses by preventing the production of antiviral cytoplasmic stress granules [25]. Nsp15 also has an essential role in the replication of corona-viruses in processing of the viral genome [14,26, 27]. Nsp15 deletion suppresses viral replication substantially [23,25, 28]. Thus, inhibiting Nsp15 activity at the non-symptomatic stage may be a potential strategy for preventing and controlling viral infection by interrupting viral genome replication and activating the host innate immune response [29]. The SARS-CoV-2 Endo RNase (nsp15) contains 345 amino acids which are located in the region of ORFlab polyprotein, ranging from 6453 to 6798 amino acids. Therefore, it is of interest to document the the potential effects of mutations on the activity of nsp15 protein from Asian SARS-CoV-2 and compare it with those of Wuhan isolates. Considerable alterations in its structural conformations have been observed. Nsp15 plays pivotal role in the life cycle of SARS-CoV-2 hence; it can be used as a major antiviral therapeutic target.

## Methods:

### Sequence retrieval:

nsp15 protein sequences of SARS-CoV-2 were retrieved from NCBI virus database, submitted from Asia from the onset of this disease till 31st January, 2022. A reference sequence of Wuhan type virus with accession number YP_009724389 was used for identification of nsp15 mutants.

### Identification of nsp15 mutants:

The nsp15 protein sequences (345 amino acids residues) were downloaded and aligned using Clustal Omega online server and the alignment was viewed using Jalview. The differences occurring in the nsp15 protein sequence with respect to Wuhan type virus sequence was noted. The frequency of different mutations was calculated.

### Calculation of physicochemical properties and hydropathy index of nsp15 protein:

The physicochemical properties including molecular weight, amino acid composition, overall pH, instability index, aliphatic index, extinction coefficient, half life and average of hydrophobicity (GRAVY) was estimated using Protparam tool of Expasy online program. Hydropathy plot of nsp15 protein was prepared using Protscale tool of expasy [30].

### Prediction of 3D structure of nsp15 protein:

The 3D structures of both the wild type and mutated nsp15 proteins were built using Chimera software [31]. Chimera is a tool for visualization of and analysis of molecular structures.

### Prediction of secondary structure and Ramachandran plot:

To know the number of alpha helices, beta sheets and turns, the secondary structure prediction of the wild type nsp15 protein was done using CFSSP (Chou and Fasman secondary structure prediction) online software [32]. Ramachandran plot of nsp15 protein was predicted using Swiss model software.

### Identification of linear B-cell epitopes:

The linear B-cell epitopes of the nsp15 protein were predicted using IEDB webserver [33] which identifies B-cell epitopes based on properties like flexibility, accessibility, hydrophilicity, turns, polarity and antigenic propensity of the protein using amino acid scales and HMMs.

## Results and Discussion:

A total of 7793 ORF1ab full length protein sequences were submitted from Asia till 31st January, 2022 from onset of this pandemic. These sequences were downloaded along with a reference sequence of Wuhan type virus from NCBI virus database. The alignments were observed using Jalview to mark the differences occurring in the nsp15 region. A total of 1795 point mutations were detected in the nsp15 protein region of ORF1ab polyprotein amongst the sequences. Amongst these point mutations, A231V, H234Y, K109N, K259R and S261A were the most frequently occurring mutations and hence used for further characterization in this study (Figure 1 - see PDF). The estimation of physicochemical properties of nsp15 protein revealed that nsp15 protein is 345 amino acids long with a molecular weight 38813.40, aliphatic index 95.09, instability index 36.28, and GRAVY score of -0.076 (Table 1 - see PDF). The hydropathy plot showed C-terminal amino acid to be more hydrophobic as compared to the N-terminal end of nsp15 protein (Figure 2 - see PDF). The nsp15 protein sequence was modeled using Chimera software. The models of both wild type and mutated protein sequences were built as shown in Figure 3(see PDF). Ramachandran plot analysis showed that most of the amino acid sequences were in the favoured region of the plot (Figure 4 - see PDF). Secondary structure analysis was done using CFSSP online program to detect the number of alpha helix, beta sheet and turns in nsp15 protein which is shown in Figure 5(see PDF).

A total of nine linear B-cell epitopes were predicted for 345 amino acid long nsp15 protein as shown in Figure 6 (Table 2 - see PDF). These epitopes can induce antibody production and hence play a crucial role in hummoral immunity. Coronavirus poses humanitarian health crisis globally. Considering its infectivity, World Health Organization on 11th March 2020 has declared public health emergency internationally (WHO 2020). SARS-CoV-2 belongs to RNA viruses family and has remarkable capacity to mutate their genome in a very short span of time [34-35]. Notably, majority of viral mutation shows harmful effects, moreover, a mutation is essential for viral evolution and adaptability, these traits are found as the key determinants for these viruses to better survive in the dynamic host environment. The findings of the present study, emphasizes mainly on the occurrence of recurrent mutations in the nsp15 protein of Asian SARS-CoV-2 isolates; by comparing it with that of Wuhan SARS-CoV-2 isolate. Nsp15 is indispensable for viral RNA cleavage and also involved in the process of pathogenicity. The results of multiple sequence alignment revealed the presence of 1795 mutations in the SARS-CoV-2 nsp15 protein, out of which A231V, H234Y, K109N, K259R and S261A occurred most frequently as shown in Figure 1(see PDF). The Ramachandran plot analysis showed the presence of major amino acids in the favored region of the quadrate. Prediction of secondary structure showed the presence of helices and sheets in the wild type protein. Protein modeling of wild type as well as mutated protein showed significant changes in the wild type nsp15 protein upon mutation. B-cell epitope prediction confirmed the efficacy of nsp15 protein to induce humoral immune response. Therefore, these mutations might induce conformational changes and considered as major challenges in the designing of vaccine to curb SARS-CoV-2 infections. Data suggests that the nsp15 of SARS-CoV-2 is an important protein for coronavirus replication and can be used as potential antiviral solution. Earlier studies have shown that the viral nsp15 appears as challenging druggable targets. Interestingly, nsp15 protein induces humoral immunity. Therefore, SARS-CoV-2 nsp15 is a suitable target for anti-viral drug discovery, as there is paucity of available information regarding designing of SARS-CoV-2 nsp15 inhibitors [36, 37].

## Conclusion:

Data provides insights into impact of mutation in developing antiviral drugs to curb this pandemic because these mutations might have functional consequences which need to be incorporated in ongoing future research work.
